# Fatty acid kinase A is an important determinant of biofilm formation in *Staphylococcus aureus* USA300

**DOI:** 10.1186/s12864-015-1956-8

**Published:** 2015-10-26

**Authors:** J. S. Sabirova, J-P Hernalsteens, S. De Backer, B. B. Xavier, P. Moons, A. Turlej-Rogacka, H. De Greve, H. Goossens, S. Malhotra-Kumar

**Affiliations:** Laboratory of Medical Microbiology, Vaccine and Infectious Disease Institute, University of Antwerp, Antwerp, Belgium; Viral Genetics Research Group, Vrije Universiteit Brussel, Brussels, Belgium; Structural Biology Brussels, Vrije Universiteit Brussel, Brussels, Belgium; Structural and Molecular Microbiology, Structural Biology Research Center, VIB, Brussels, Belgium; Department of Medical Microbiology, Campus Drie Eiken, University of Antwerp, S6, Universiteitsplein 1, B-2610 Wilrijk, Belgium

**Keywords:** *vfrB*, *fakA*, MRSA, Methicillin-resistant *S. aureus*, Transposon mutagenesis, Whole genome sequencing, SNPs, USA300-UAS391, USA300_UAS391, USA300, UAS391

## Abstract

**Background:**

Methicillin-resistant *Staphylococcus aureus* (MRSA)-USA300 is notorious for its ability to cause community- and healthcare-acquired infections, which are even more difficult to treat when associated with a biofilm phenotype. We aimed to characterize the genetic determinants of biofilm formation in a USA300 skin abscess isolate (UAS391) that formed prolific biofilms.

**Methods:**

USA300 *S. aureus* strains, TCH1516 and FPR3757, were found to be closely related based on whole genome mapping (Argus™ Optical Mapping System, Opgen Inc, Gaithersburg, USA) to UAS391 (96.3-99.1 % similarity, P=0.0151), however differed markedly in biofilm formation (P=0.0001) on a dynamic assay (BioFlux 200, Fluxion Biosciences, USA). Comparison of whole genome sequences of these strains identified differences in a total of 18 genes. Corresponding Tn (*bursa aurealis*-bearing) knockout mutants in these target genes were obtained from a publicly available mutant library of the same clonal lineage (USA300-JE2) and were characterized phenotypically for biofilm formation. Tn mutants showing significant differences in biofilm formation were utilized for transduction into a plasmid-cured erythromycin-sensitive derivative of UAS391 and for complementation experiments. All strains were tested on the dynamic assay, and 17h-biofilms were stained (SYTO9, Life Technologies) and fluorescence intensity quantified by microscopy (Zeiss, ImageJ). Gene expression levels in Tn and transduced mutants were studied by quantitative reverse transcriptase PCR (StepOnePlusTM, Applied Biosystems®).

**Results:**

Comparison of the sequenced genomes of TCH1516, FPR3757 and UAS391 yielded a limited number of variant genes (n=18) that were hypothesized to account for the observed difference in biofilm-forming capacity. Screening of Tn mutants disrupted in these target genes identified one mutant (NE229) bearing a transposon insertion in SAUSA300_1119 (*fakA*), which exhibited increased biofilm formation similar to UAS391 (P=0.9320). Transduction experiments confirmed that *fakA*::Tn corresponded to 1.9- to 4.6-fold increase in biofilm formation depending on the USA300 strain background (P≤0.0007), while complementation of the TCH1516 wild-type *fakA* allele in UAS391 resulted in a 4.3-fold reduction in biofilm formation (P<0.0001).

**Conclusions:**

This sequential approach, consisting of strain typing, genome comparison and functional genomics, identified *fakA*, a recently described fatty acid kinase in *S. aureus* that is essential for phospholipid synthesis and also impacts the transcription of numerous virulence factors, as a negative regulator of biofilm formation in *S. aureus* USA300.

**Electronic supplementary material:**

The online version of this article (doi:10.1186/s12864-015-1956-8) contains supplementary material, which is available to authorized users.

## Background

*Staphylococcus aureus* causes infections ranging from minor skin infections to life-threatening diseases, such as pneumonia, meningitis, osteomyelitis, endocarditis, and septicaemia. Since their emergence in the 1960s, methicillin-resistant *S. aureus* have become one of the major causes of hospital-acquired (HA) infections such as implant-associated and postsurgical wound infections, as well as of community-acquired (CA) infections such as pneumonia. The success of these hospital-acquired MRSA (HA-MRSA) clones can be partly attributed to virulence-specific factors, such as extracellular toxins, surface structures facilitating tissue colonization, immune evasion and tissue destruction [1], as well as to prolonged persistence of MRSA infections linked to the formation of biofilms *in vivo* [2].

It is generally accepted that biofilms, comprising conglomerations of cells attached to a solid support and embedded in a matrix of extracellular polymers, represent a major problem in clinical practice, due to their formation on implanted medical devices [3] and their intrinsic enhanced resistance to antibiotics that are otherwise efficacious against the bacterium’s planktonic life forms [4]. These biofilm-associated complications have triggered the search for potential genes and/or metabolic pathways, interruption of which could represent new therapeutic or preventive interventions specifically targeting this bacterial life-style. Functional genomics approaches such as transcriptomics and proteomics, performed on biofilm versus planktonic cells, have shed some light on the complexity of the biofilm phenotype in *S. aureus*. Among genes found to be up-regulated in biofilm cells were those encoding proteins involved in the synthesis of polysaccharide intercellular adhesin (PIA)/ polymeric *N*-acetyl-glucosamine (PNAG) as well as proteins mediating transport, amino acid metabolism and translation, with many other up-regulated genes encoding hypothetical proteins of still unknown function [5]. Subsequent proteomic studies performed by Resch et al. [6] showed still more stringently that biofilm-induced proteins are involved in cell attachment and peptidoglycan synthesis, in pyruvate and formate metabolism, as well as in regulatory processes, in particular those exerted by the staphylococcal accessory regulator A protein.

We recently screened a large collection of clinically important MRSA isolates for their ability to form biofilms, and subsequently typed these isolates [7]. This work yielded a number of strong biofilm-forming strains, with three of them belonging to the USA300 clonal lineage, one of which was selected for genome sequencing. Subsequent comparison of the strain’s genome with those of other USA300 sequenced isolates revealed a particularly interesting pair of closely related USA300 strains showing notably different capacities for biofilm formation (UAS391 and USA300_TCH516). This finding offered the opportunity to search in clinical isolates at genome level for mechanisms of biofilm formation. Thus, various USA300 *S. aureus* clones, which are closely related as per whole genome mapping, but markedly different in biofilm formation, were compared at genome level and consequently genetic loci with different alleles were identified. Corresponding knockout mutants of these genes, obtained from a publically available mutant library of the same clonal lineage (USA300-JE2), were then phenotypically characterized for their potential role in biofilm formation. Transposon-mediated interruption of one of the tested divergent genes, SAUSA300_1119, corresponding to the gene *fakA*, resulted in drastically increased levels of biofilms as compared to the parental control strain JE2, marking this gene as an important determinant of biofilm formation. These results shed more light on the genetic factors regulating biofilm formation in *S. aureus*.

## Methods

### Bacterial strains and growth conditions

USA300 strains used for clonal, genomic, and phenotypic analyses are listed in Table 1. Tn insertion mutants used for genotypic and phenotypic analysis of biofilm formation, were obtained from a sequence-defined transposon mutant library consisting of 1,952 strains, each containing a single mutation within a nonessential gene in strain USA300 JE2 [8] obtained from the NARSA repository (www.beiresources.org/). *S. aureus* USA300 FPR3757 (FPR3757) and USA300 JE2 (JE2) were also obtained from the NARSA repository. Strains UAS391, TCH1516, FPR3757 and JE2 were routinely grown on brain heart infusion (BHI) and lysogeny broth (LB) medium for biofilm and transduction experiments, respectively. Transposon insertion mutants were grown on BHI medium containing 10 μg/ml of erythromycin (Sigma-Aldrich).

**Table 1:** Strains and plasmids used for clonal, genomic, and phenotypic analyses during this study

Strains/Plasmids	Description	Source
Strains		
UA S391	*S. aureus* strain USA_UAS391	[18]
JE2	*S. aureus* USA300 parental strain for the NARSA transpos on library	NARSA^a^
FPR3757	*S. aureus* USA300_FPR3757	NARSA
TCH1516	*S. aureus* subsp*. aureus* USA300_TCH1516	ATCC
UA S391-Ery^s^	Heat-cured erythromycin-sensitive derive of UAS391	This study
TCH1516-Ery^s^	Heat-cured erythromycin-sensitive derive of TCH1516	This study
UA S391-NE229	UA S391-Ery^s^ transductant with mutation in EX97_05885	This study
TCH1516-NE229	TCH1516-Ery^s^ transductant with mutation in USA 300Hou_1162	This study
NE229-pHD954	*S. aureus* stra NE229 completed with pHD954	This study
NE229-pHD957	*S. aureus* strain NE229 completed with pHD957	This study
UA S391-pHD957	*S. aureus* strain UA S391 completed with pHD957	This study
RN0451	Phage Φ11 lysogenic *S. aureus* strain (NARSA strain NRS 136)	NARSA
RN0450	*S. aureus* RN0450 (NARSA strain NRS 135)	NARSA
RN4220	Restriction-deficient intermediate *S. aureus* cloning host RN4420	NARSA
	Restriction-deficient intermediate *S. aureus* cloning host RN4420	NARSA
NE1646	Transposon mutant with insertion in USA200HOU_0155 (SA USA300_0145)	NARSA
NE229	Transpos on mutant with insertion in USA300HOU_1162 (SA USA300_1119)	NARSA
NE1081	Transpo on mutant with insertion in USA300HOU_2626 (SA USA300_2561)	NARSA
NE454	Transpo on mutant with insertion in USA300HOU_2631 (SA USA300_2566)	NARSA
NE1290	Transposon mutant with insertion USA300HOU_2641 (SA USA300_2576)	NARSA
NE81	Transposon mutant with insertion in USA300HOU_2319 (SA USA300_2285)	NARSA
NE1036	Transposon mutant with insertion in USA300HOU_2602 (SA USA300_2542)	NARSA
NE1038	Transposon mutant with insertion in USA300HOU_2678 (SA USA300_2610)	NARSA
NE33	Transposon mutant with insertion in USA300HOU_2654 (SA USA300_2589)	NARSA
NE1262	Transposon mutant with insertion in USA300HOU_2026 (SA USA300_1984)	NARSA
NE1875	Transposon mutant with insertion in USA300HOU_1934 (SA USA300_1918)	NARSA
NE1	Transposon mutant with insertion in USA300HOU_1372 (SaA USA300_1327)	NARSA
NE334	Transposon mutant with insertion in USA300HOU_2197 (SA USA300_2161)	NARSA
NE809	Transposon mutant with insertion in USA300HOU_1260 (SA USA300_1214)	NARSA
NE1403	Transposon mutant with insertion in USA300HOU_1260 (SA USA300_1585)	NARSA
NE1026	Transposon mutant with insertion in USA 300HOU_1338 (SA USA 300_1298)	NARSA
NE1314	Transposon mutant with insertion in USA300HOU_0953(SA USA300_0896)	NARSA
Plasmids		
pALC2073	Shuttle vector pALC2073	[11]
pHD954	pALC2073 with cloned *fakA* gene amplifie from UAS391	This study
pHD957	pALC2073 with cloned *fakA* gene amplifie from TCH15116	This study

^a^This library now exists at www.beiresources.org and is discussed by Fey et al. [8]

### Transduction experiments

Transduction was performed essentially as described [9]. Transducing phage ϕ11 was obtained from the supernatant of a culture of the lysogenic *S. aureus* strain RN0451 (NARSA strain NRS136) and propagated on *S. aureus* RN0450 (NARSA strain NRS135) by standard techniques [10]. A transducing phage stock was prepared by infection at 37 °C of *S. aureus* containing the *bursa aurealis* transposon insertion. After infection of cultures of the recipient *S. aureus* strains (UAS391 and TCH1516) with this stock, transductants were selected on LB plates with 0.05 % sodium citrate containing 5 mg/L erythromycin at 37 °C. The resulting colonies were purified at least twice on the same medium to ensure loss of the transducing phage.

### Complementation experiments

To complement the *S. aureus* mutant strain NE229, total genomic DNA of strains UAS391 and TCH1516 was purified with the Quick Pick™ SML gDNA kit (BN Products & Services) according to the manufacturer’s recommendations. *S. aureus* strains were lysed by adding 5 μg lysostaphin (Sigma-Aldrich). The genes corresponding to SAUSA300_1119 from the *S. aureus* strains UAS391 and TCH1516 were amplified using ExTaq DNA polymerase (Takara) with the primer pair Glyk-1 (5’-TACCGAGCTCGAATTCTAGGAGGACAACTTGAAATGATTAG-3’) and Glyk-2 (5’-GACGGCCAGTGAATTCATTTTTATTCTACTGAAAAGAAATATTG-3’). Polymerase chain reactions (PCR) were carried out in an Applied Biosystems 2720 Thermal Cycler using Ex Takara DNA Polymerase. Annealing and elongation temperatures were 55 °C and 68 °C respectively, with an elongation time of 1 min per 1000 bp. during 30 cycles. PCR-fragments were purified using the Qiaquick PCR Purification Kit (Qiagen GmbH) and analysed by gel electrophoresis on 1.0 % agarose gels. The resulting 1753 bp. PCR fragments were cloned by the InFusion technique (Clontech Laboratories, Inc) in the *EcoRI* site of the shuttle vector pALC2073 [11] yielding the plasmids pHD954 (UAS391) and pHD957 (TCH1516). DNA sequencing was performed at the VIB core sequencing facility (VIB Genetics Department, University of Antwerp) using the pALC2073 vector primers TetR2 (5’-CAATGTAGGCTGCTCTACACCTAG-3’), pALC-2 (5’-GATCGGTGCGGGCCTCTTCGCTAT-3’), and the internal gene sequence primers Glyk-3 (5’-GGAGTACATTATTGTAAAAGCCAATGAATC-3’) and Glyk-4 (5’- CCACACATATCATTAGTGGTGGACA-3’). These plasmids were transferred into the restriction-deficient intermediate *S. aureus* cloning host RN4220 to adapt the plasmid DNA [12] to the *S. aureus* modifications. Transformants were selected on LB plates supplemented with 10 μg/ml chloramphenicol (Sigma-Aldrich). The plasmids pHD954 and pHD957 were isolated from the RN4220 strain and used to transform strain NE229. UAS391 was also complemented with the wild-type *fakA* allele carried on pHD957.

### RNA extraction and RT-PCR

Mutant strain NE229 and wild-type strains FPR3757 and JE2 were grown as overnight cultures in 10 ml of BHI medium at 37 °C with shaking. Total RNA of the three bacterial strains was extracted after 16 h growth using Express Amptec kit (Ambion). Two μg of RNA was treated with DNase using the Turbo DNA-free™ Kit (Ambion® by Life Technologies™) and subsequently used for reverse transcription reaction using the Reverse Transcription System (Promega) with random primers according to the manufacturer’s instructions. Real-time PCR was performed using a StepOnePlus™ system (Applied Biosystems®) in a 20 μl reaction mixture with Absolute Blue qPCR SYBR green ROX mix (Thermo Scientific, Inc). For RT-PCR analysis, the cDNA samples were amplified with gene-specific primers 1162 F (5’-ATGATGTGGACGCAACACTTG-3’) and 1162Rev (5’-AATCAAGCCCATAAACGCGTC-3’), in duplicate. Cycling conditions were 95 °C for 10 min, and 40 cycles of 95 °C for 15 s and 60 °C for 60 s and 72 °C for 40 s. Amplification plot and melting curve were analysed for the dynamics of fluorescence and specificity of amplification, correspondingly. Resulting PCR products were checked by gel electrophoresis.

### Whole genome mapping

For WGM, UAS391 and TCH1516 were grown on BHI plates, and high molecular weight DNA was extracted from the overnight colonies using Argus® HMW DNA extraction kit (Opgen, Inc). Following DNA extraction as recommended by manufactures protocol, DNA molecules were loaded on a MapCard surface (Opgen, Inc) where single DNA molecules were immobilized and linearized. The linearized DNAs were subjected to *in situ* digestion with *NcoI* (Opgen, Inc). Following digestion, the DNA molecules were stained with the fluorescent intercalating agent JoJo-1 forming part of the staining kit (Opgen, Inc). The digested and stained DNA fragments were imaged and assembled by in built assembler of Map Manager to produce whole genome restriction maps, as described previously [13]. Finally, derived whole genome maps were analysed using BioNumerics v7.5. (Applied Maths, Belgium).

### Genome sequencing

The complete genome sequence of UAS391 was generated by using the Illumina HiSeq2000 platform, as described previously [14]. Sequence data of UAS391 were *de novo* assembled using Velvet [15] and SPAdes [16]. Assembled contigs were ordered against the UAS391 whole genome map using MapSolver software (Opgen, Inc). Validated scaffolds were ordered against published *S. aureus* genome TCH1516 (accession no. CP000730), and the generated pseudo chromosome was compared to the genomes of TCH1516 and FPR3757 (accession no. CP000255.1) using Mauve v2.3.1 [17]. Similarly, we also performed reference assembly independently by using the genome sequences of TCH1516 and FPR3757 as references, and SNPs were extracted in CLC Genomics Workbench 7.5.1 (QIAGEN, Aarhus A/S, Denmark). The assembled chromosome of UAS391 was annotated as described [18]. Multiple alignment and phylogenetic analysis were performed using MEGA6 [19].

### Flow biofilm assay and quantification of biofilm mass

A medium-throughput continuous flow system BioFlux 200 (Fluxion Biosciences, USA) was used to study biofilm formation under shear flow conditions, which mimics flow conditions of physiological liquids in the human body [20]. BHI or 0.5xBHI with 0.1 % glucose was used to feed into the flow cell. Bacterial cultures at 0.05 MacFarland were then used to inoculate the output wells; bacteria were pushed through the flow cell from the output well up to the horizontal microfluidic channel by reversing the flow and were allowed to attach for one hour followed by 16 h of incubation at 37 °C in BHI or 0.5xBHI with 0.1 % glucose at a flow rate of 0.5 dyne/cm^2^. Biomass in the microfluidic channels was stained with SYTO 9 fluorescent stain (Invitrogen, Life Technologies). Biofilm images were captured employing ZEN 2012 software (Zeiss) as combined tile images consisting of 81 one μm^2^ horizontal tiles covering the entire microchannel. Actual fluorescence quantification was recorded as integrated density on the entire combined tile image using Image J freeware (http://imagej.nih.gov/) using “integrated density”, “mean value” and “area” as measurement settings. Presented values are averages of three independent combined tile images.

### Statistical analysis

Statistical analysis of biomass formation in the dynamic flow assay was performed using the R Project software (version 3.1.2.). The influence of a particular gene interruption on biofilm formation and the comparison of the space occupied by the cells within the dynamic biofilms were analysed using a pairwise one-way ANOVA, Shapiro-Wilk normality testing, Bartlett’s test of variances, and Tukey’s honest significance difference testing. Results from RT-PCR were analysed through a Wilcoxon signed-rank test. *P* < 0.05 were considered to be significant.

## Results

### Typing and phenotypic analysis of USA300 isolates

**Fig. 1:**
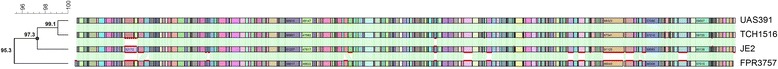
Whole genome maps of USA300 strains. Green lines indicate identity of restriction pattern among the maps and red horizontal marks represent the variations

**Fig. 2:**
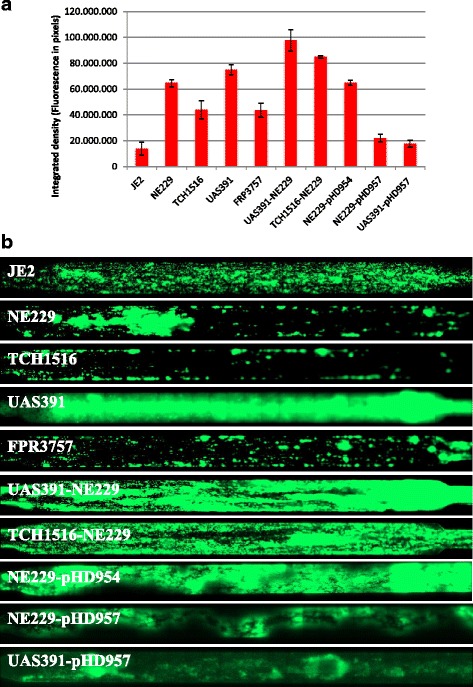
Biofilm formation of USA300 strains in the dynamic shear flow assay. Quantification (**a**) and visualization (**b**) of biofilms formed by wild-type USA300 strains and corresponding derivate strains

Whole genome mapping (WGM) allowed typing and grouping of strain UAS391 with sepsis strain USA300_TCH1516 (TCH1516) isolated at Texas Children’s Hospital in Houston [21] along with two other *S. aureus* USA300 strains, USA300-FPR3757 (FPR3757) and its plasmid-cured laboratory derivative USA300-JE2 (JE2), both belonging to the USA300 clonal lineage. According to the WGM similarity cut-off recently established for USA300 isolates [22], a WGM-based clonal cluster is defined as a set of isolates having a whole genome map similarity of >95 %, which assigned all four isolates discussed here to the same WGM clonal cluster (Fig. 1). As UAS391 was previously identified as a prolific biofilm former [7], we directly compared its biofilm forming capacity to the other clonally related strains in a dynamic biofilm assay. Comparison of UAS391 with the other USA300 isolates in this dynamic biofilm model consistently revealed clear differences in this strain’s ability to form biofilms under these conditions, with UAS391 forming 1.6- to 3.6-fold more biofilm than the other three USA300 strains, as measured by readout of fluorescence (*P* < 0.0001, ANOVA, F = 56.95, df = 3) (Fig. 2).

### Identifying potential gene targets by comparative genomics

Genomic divergence between phenotypically diverse strains from the USA300 clonal lineage should by definition be very limited [21, 23]. Since TCH1516 was closely related to UAS391 as per WGM and its total genome sequence is already available [21], we reasoned that comparing the whole genome sequences of UAS391 and TCH1516 would likely yield genetic differences that could account for the observed differences in the biofilm phenotype between these two USA300 strains. We thus sequenced the UAS391 genome by using the Illumina HiSeq2000 platform [18], and identified a total of 52 gene loci where UAS391 and TCH1516 exhibited single nucleotide polymorphisms (SNPs) (Additional file 1: Table S1), of which 47 mapped within open reading frames (ORFs) and 5 within intergenic regions.

Based on genome sequence data and comparison of whole genome maps (Fig. 1), FPR3757 is closely related to TCH1516 [24], and thus also to UAS391, and forms less biofilms in the dynamic biofilm model, compared to UAS391 (*P* = 0.0172) and similar to TCH1516 (Fig. 2) (*P* = 1.0000). As the complete sequence for FPR3757 is also available, we then compared UAS391 and FPR3757 and identified a total of 91 gene loci where UAS391 and FPR3757 exhibited SNPs (Additional file 2: Table S2), of which 60 mapped within ORFs and 31 within intergenic regions. To further narrow down this initial list of deviating genes, we identified an overlap between the gene alleles of UAS391, TCH1516 and FPR3757 (Additional file 1: Table S1 and Additional file 2: Table S2). This reduced the initial list of genetic variations between strains UAS391 and TCH1516 to only those SNPs that were identical in the two weak biofilm forming strains TCH1516 and FPR3757 but different in UAS391, potentially identifying those SNPs primarily responsible for the increased biofilm formation in UAS391. This comparison yielded 18 SNPs as shown in Table 2. Of this, SNPs in transposases and phage-elements (USA300HOU_0286 and 1488) were excluded from further analysis (*n* = 2).

**Table 2:** Single nucleotide polymorphisms (SNPs) between TCH1516/FPR3757 and UAS391. Consensus base represents TCH1516 and FPR3757, allele represents a corresponding base in UAS391. Predicted amino acid changes in UAS391 compared to TCH1516 and FPR3757 were not displayed for synonymous SNPs

Consensus base (TCH1516/FPR3757)	Allele UAS391	Gene locus tag (in TCH1516/FPR3757)	Predicted amino acid change (TCH1516/FPR3757)	Gene and putative function
G	T	USA300HOU_0286/SAUSA300_0267	Leu1Phe	IS1272 transposase
T	C	USA300HOU_0155/SAUSA300_0145		Phosphate-import protein PhnD/ABC Transporter, periplasmic binding protein
G	A	USA300HOU_0521/SAUSA300_0513	Met386Ile	*gtlX*: glutamyl-tRNA synthetase
A	C	USA300HOU_1051/SAUSA300_1013	Asn267Thr/Asn263Thr	Bacterial cell division membrane protein FtsW
A	G	USA300HOU_1162/SAUSA300_1119	Glu524Gly	*fakA*: Fatty acid kinase
G	T	USA300HOU_1166/SAUSA300_1123	Gly169Val	*fabD*: malonyl CoA-acyl carrier protein transacylase (Lipid metabolism)
A	G	USA300HOU_1372/SAUSA300_1327	Val1114Ala	Ebh: Cell Wall-Associated Fibronectin-Binding Protein
C	A	USA300HOU_2197/SAUSA300_2161		Hyaluronate lyase is a glycosaminoglycan (GAG) polysaccharide lyase family. This family consists of a group of secreted bacterial lyase enzymes capable of acting on glycosaminoglycans, such as hyaluronan and chondroitin, in the extracellular matrix of host tissues, contributing to the invasive capacity of the pathogen.
G	A	USA300HOU_2631/SAUSA300_2566	Pro27Ser	HTH-type Transcriptional regulator ArcR, signal transduction system
C	T	USA300HOU_2641/SAUSA300_2576		PTS system, fructose-specific II ABC component, multi protein system involved in regulation of metabolic and transcriptional process
T	C	USA300HOU_2319/SAUSA300_2285		Aldose 1-epimerase
G	A	USA300HOU_2602/SAUSA300_2542	Arg517* ^1^	Acyl-coenzyme A synthetases/AMP-(fatty) acid ligases (Lipid metabolism)
A	C	USA300HOU_2654/SAUSA300_2589	Ser1782Ala	Serine-rich adhesin for platelets, cell surface protein precursor; KxYKxGKxW signal peptide
C	T	USA300HOU_0502/SAUSA300_0486	Thr85Met	Uncharacterized protein YabR
C	T	USA300HOU_1626/SAUSA300_1585	Ser35Leu	tRNA threonylcarbamoyladenosine dehydratase (Cells lacking this gene have a normal growth phenotype, but are unable to survive in a competitive growth situation with the wild-type strain. They display only the t6A but not the ct6A modification in tRNAs, and have lower decoding efficiency than wild-type. They show no defects in antibiotic sensitivity. In growth competition experiments, a *tcdA* mutant shows reduced fitness compared to wild-type, but outcompetes a *csdA* mutant.)
A	G	USA300HOU_2026/SAUSA300_1984	Lys118Arg	Putative membrane peptidase YdiL
T	C	USA300HOU_1488/SAUSA300_1436		phage lipoprotein
T	-	USA300HOU_1338/SAUSA300_1298	Phe130fs^2^	5-bromo-4-chloroindolyl phosphate hydrolysis protein

^1^The symbol (*) refers to the predicted amino acid change as a stopcodon caused by the corresponding SNP

^2^The abbreviation (fs) refers to ‘frame shift mutation’

Similarly, comparative genome alignment was performed for TCH1516, FPR3757 and UAS391 to confirm the data obtained by whole genome sequencing (Additional file 3: Figure S1).

### Identification of genes involved in biofilm formation using gene knockout mutants

**Fig. 3:**
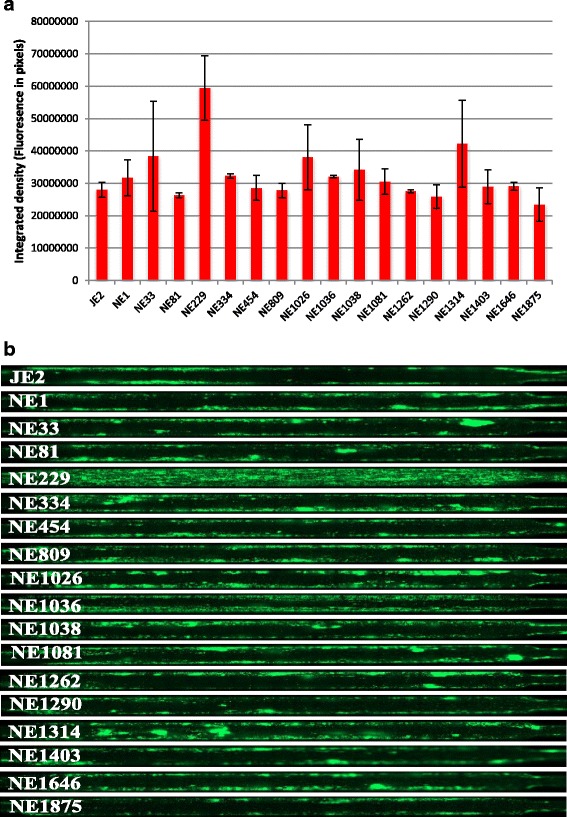
Biofilm formation of USA300 strains in the dynamic shear flow assay. Quantification (**a**) and visualization (**b**) of biofilms formed by JE2 and its transposon mutants

In order to investigate the role of the corresponding genes in biofilm formation, we obtained 12 mutants from the Network on Antimicrobial Resistance in *S. aureus* (NARSA) library in which transposon insertions map in genes affected by SNPs, belonging to the above identified group of 16 genes enumerated in Table 2. For the remaining four candidate genes, USA300HOU_0521, 1051, 1166, and 0502, corresponding knockout mutants were not present in the library and these were not studied further. We also randomly selected five genes affected by SNPs solely in either TCH1516 or FPR3757 in comparison to UAS391. In total, the 17 mutants obtained from the NARSA library (corresponding to USA300HOU_ 0953, 0155, 1162, 1260, 1372, 2197, 2626, 2631, 2641, 2319, 2602, 2678, 2654, 1626, 2026, 1338, 1943) were phenotypically tested in duplicate for their ability to form flow biofilms using the library parental strain JE2 as the corresponding control. Significant positive and negative variations of the degree of biofilm formation as compared to JE2 were found (*P* = 0.0018, ANOVA, F = 4.44, df = 17). Out of the 17 mutants tested (Fig. 3), one mutant NE229 showed a 2-fold increase in biofilm formation compared to JE2 (*P* = 0.0024). NE229 harbours the transposon insertion at position 393 in ORF SAUSA300_1119 encoding the fatty acid kinase *fakA* located in the genome from position 1223940 to 1225586 nt [25] (Additional file 4: Figure S2).

**Fig. 4:**
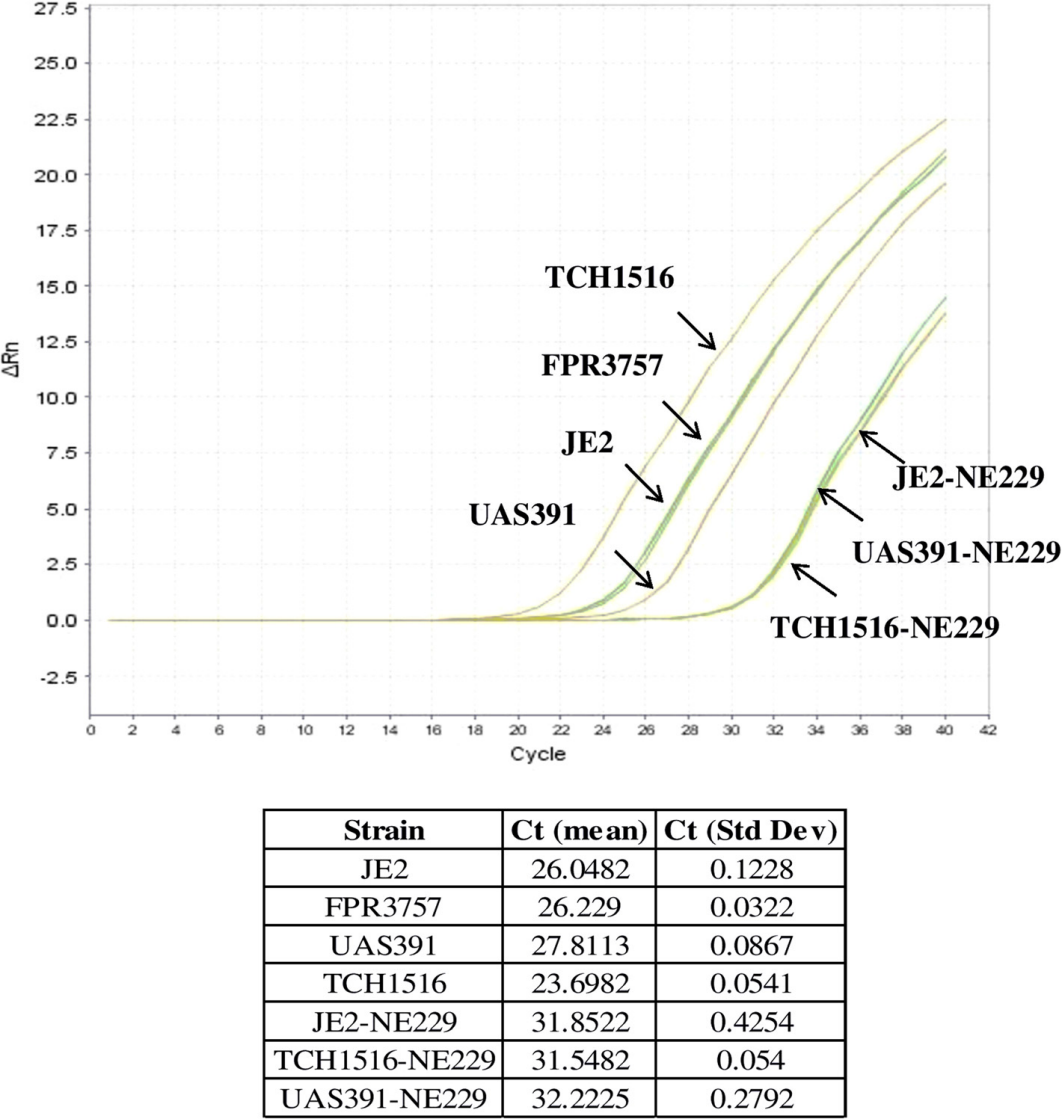
Transcription levels of the *fakA* gene in UAS391, TCH1516, FPR3757, JE2 and in corresponding transductant strains. Transcription levels of *fakA* were measured in duplicate by real-time RT-PCR and expressed as an amplification plot and Ct mean values

Reverse transcription PCR (RT-PCR) was performed to determine whether the *bursa aurealis* insertion had affected transcription of SAUSA300_1119. Primers were designed downstream of the transposon mutation. Transcription of SAUSA300_1119 in NE229 showed a significant decrease compared to that in wild-type strains FPR3757 and JE2 (Cts of 31.9, 26.2, and 26.0, respectively) (*P* < 0.0001, ANOVA, F = 1599.54, df = 6). Similar decrease in transcription was also observed for the TCH1516-EryS and the UAS391-EryS strain transduced with the SAUSA300_1119 mutated allele from NE229 (*P* < 0.0001) (Fig. 4).

The SAUSA300_1119 in UAS391 harbours a non-synonymous SNP (Glu524Gly) as compared to strains TCH1516 and FPR3757 (Table 2). BLAST search identified it as a unique SNP and it can thus be considered as the mutant version of the corresponding gene present in TCH1516 and FPR3757, with similar wild-type alleles being present among other sequenced *S. aureus* isolates (Additional file 4: Figure S2).

### Transductants mutated in fakA gene exhibit elevated biofilm formation

In order to further corroborate the negative role of *fakA* in biofilm formation by USA300, the knockout mutation in gene SAUSA300_1119 was transferred from NE229 into both UAS391-EryS and TCH1516-EryS employing phage-mediated transduction resulting in UAS391-NE229 and TCH1516-NE229, respectively. Knocking out SAUSA300_1119 in TCH1516 (locus tag USA300HOU_1162) resulted in a 1.7-fold increase in biofilm formation compared to the wild type TCH1516 (*P* = 0.0007), and at levels similar to the wild-type UAS391 (*P* = 0.9710) (Fig. 2). This effect was also observed in the UAS391 background where UAS391-NE229 exhibited even more abundant biofilm formation than the wild-type UAS391 (*P* = 0.0510) (Fig. 2).

### A plasmid-borne copy of the wild-type fakA gene complements the mutant phenotype

In order to unambiguously prove that the enhanced biofilm phenotype observed in the NE229 strain is indeed due to the knockout mutation of the *fakA* gene, the mutant strain was complemented with a plasmid-borne intact *fakA* allele present in the TCH1516 strain or with the corresponding SNP-containing allele present in the UAS391 strain, yielding NE229-pHD957 and NE229-pHD954 strains, respectively. As shown in Fig. 2, the amount of biofilm produced by NE229-pHD957 was 2.9-fold lower as compared to NE229 (*P* = 0.0003), and similar to the amount of biofilm produced by the parent JE2 strain (*P* = 0.9831), whereas the amount of biofilm produced by NE229-pHD954, containing only mutant *fakA* allele was the same as in NE229 (*P* = 1.0000). Additionally, complementing UAS391 with pHD957 resulted in a 4.3-fold decrease in biofilm mass as compared to the parent UAS391 (*P* < 0.0001).

## Discussion

By comparing the whole genome sequences of closely related USA300 strains that strongly differed in their capacity to form biofilms in a dynamic flow model, identified genetic differences (SNPs) were hypothesized to be responsible for this altered biofilm phenotype. Seventeen transposon mutants knocked out in these genes in the USA300-JE2 background (NARSA strains) were evaluated for the degree of biofilm formation in comparison to the parent JE2. Transposon-mediated interruption of one of the tested divergent genes, *fakA* [25], previously known as *vfrB* [26], resulted in a 4.6-fold increase in biofilm formation as compared to the parental control strain JE2. In the other USA300 strains, UAS391 and TCH1516, the *fakA*::Tn mutations also led to increased biofilm formation, and further complementation experiments confirmed the role of *fakA* in the regulation of biofilm formation.

Running the protein sequence of SAUSA300_1119 in Simple Modular Architecture Research Tool (SMART) identified two domains within the protein sequence: Dak2 encoding the predicted phosphatase domain of the dihydroxyacetone kinase family (35 to 200 nt), and Dak1_2 encoding the kinase domain of the dihydroxyacetone kinase family (236 nt to 548 nt). The glutamic acid to glycine change is located at position 524 of the protein corresponding to its kinase domain. These proteins, collectively called Dak2 domain proteins have homologues in a wide variety of bacteria. Transposon insertions in *fakA* were first isolated in a large *S. aureus* transposon (*bursa aurealis*-bearing) insertion mutants library screen based on an increased resistance to an antimicrobial peptide [27]. Importantly, this *fakA* (then called *dak2*) mutant exhibited an altered membrane phospholipid composition compared to its wild-type parent [27]. Recent studies have further delineated the multiple functions of *fakA* as an important regulator of virulence factors [26] and as a fatty acid kinase responsible for host fatty acid incorporation by *S. aureus* [25]. Interestingly, in the present study, complementation by the *fakA* allele of TCH1516, which is the consensus allele, in the *fakA*::Tn NE229 and in the UAS391 strains decreased the amount of biofilm formation by 2.7- and 4.3-fold, respectively, to the level observed in the JE2 wild-type strain. However, similar complementation by the UAS391 *fakA* allele, which harbours the glutamic acid to glycine change, did not affect biofilm formation in *fakA*::Tn NE229. Taken together, these results imply that the mutation in the UAS391 *fakA* might have impacted the catalytic activity of the kinase, and whether UAS391 exhibits an altered membrane phospholipid composition due to this amino acid change, which increased its biofilm forming ability, remains to be studied.

Interestingly, microarray-based gene expression data from a USA300 *fakA*::Tn mutant showed significant up-regulation of 26 and down-regulation of 19 genes [25]. The down-regulated genes included *saeP* and *saeQ* that are part of the *saeRS* two-component regulatory system and are known to regulate the activity of the *saeS*-encoded sensor histidine kinase [28]. The *saeRS* system was also recently shown to be a negative regulator of biofilm formation in *S. aureus* [29]. This study showed that a *S. aureus* Newman ∆*saeRS* strain exhibited an enhanced biofilm phenotype, similar to the *fakA*::Tn JE2-NE229 mutant and the UAS391 wild-type strains in our study. Taken together, these phenotypic and gene expression data strongly suggest a potential interaction between *fakA* and *saeRS* in negatively regulating biofilm formation in *S. aureus*. Finally, mice infected with *vfrB*::Tn (*fakA*::Tn) *S. aureus* have been shown to develop significantly larger abscess areas and dermonecrosis [26], which also reflects the increased biofilm abundance observed for the *fakA*::Tn mutant in our study.

Thus utilizing a combination of functional assays and genomics, we identified *fakA*, a known virulence factor regulator and a fatty acid kinase, as an important negative regulator of biofilm formation in *S. aureus* USA300.

## Conclusions

The sequential approach used here, starting from comparing clonally related (as per optical mapping) clinical isolates with different clinically relevant phenotypes, then comparing the respective total genome information allowed to pinpoint a gene locus, that is clearly of relevance for biofilm formation in *S. aureus*.

## Additional files

Additional file 1: Table S1. Complete list of SNPs in genes representing divergent alleles in UAS391 and TCH1516 and their putative gene functions. TCH1516 was used as a reference for comparison with the allelic change in UAS391. Nucleotide changes in bold red represent the common SNPs in both FPR3757 and TCH1516 as compared to UAS391 (also in Table 2). (XLSX 14 kb) Additional file 2: Table S2. Complete list of SNPs in genes representing divergent alleles in UAS391 and FPR3757 and their putative gene functions. FPR3757 was used as a reference for comparison with the allelic change in UAS391. Nucleotide changes in bold red represent the common SNPs in both FPR3757 and TCH1516 as compared to UAS391 (also in Table 2). (XLSX 14 kb) Additional file 3: Figure S1. Comparative genome alignment of USA300, TCH1516, FPR3757 and UAS391 shows high conservation between these genomes. Pink regions show homology and the vertical lines shows conserved blocks. The UAS391 ~13 kb genomic region (from 680369 bp to 693620 bp) is translocated to (1630711 to 1642611bp) in FPR3757. (PDF 116 kb) Additional file 4: Figure S2. Multiple alignment of *fakA* gene sequences in *Staphylococcus aureus* and *Staphylococcus epidermidis*. Unique nucleotide variation in SAUSA300_1119 of UAS391 encoding a fatty acid kinase *fakA* at position 1223940 nt, corresponding to nt 393 of the gene. The respective SAUSA300_1119 allele in UAS391 contains a non-synonymous SNP (Glu to Gly) as compared to strains TCH1516 and FPR3757. The Genbank accession numbers were indicated among these 50 strains. The first 45 strains represent *S. aureus*, the last 5 strains represent *S. epidermidis*. (PDF 897 kb)

## Abbreviations

BHI: Brain heart infusion broth medium
BLAST: Basic local alignment search tool
CA: Community-acquired
Dak2: Dihydroxyacetone kinase 2
*fakA*: Fatty acid kinase A
HA: Hospital-acquired
HA-MRSA: Hospital-acquired methicillin-resistant *Staphylococcus aureus*
LB: Lysogeny broth medium
MRSA: Methicillin-resistant *Staphylococcus aureus*
NARSA: Network on Antimicrobial Resistance in *Staphylococcus aureus*
ORF: Open reading frame
PIA: Polysaccharide intercellular adhesion
PNAG: Polymeric N-acetyl-glucosamine
RT-PCR: Reverse transcription polymerase chain reaction
SMART: simple modular architecture research tool
SNP: Single nucleotide polymorphism
Tn: Transposon
UPGMA: Unweighted-pair group method with arithmetic averages
*vfrB*: Virulence factor regulator B
WGM: Whole genome mapping
