# Supplementary figures and images for: Fatty acid kinase A is an important determinant of biofilm formation in Staphylococcus aureus USA300
Source: BMC Genomics. 2015 Oct 26;16:861. doi: 10.1186/s12864-015-1956-8 (PMC4623894; doi:10.1186/s12864-015-1956-8)

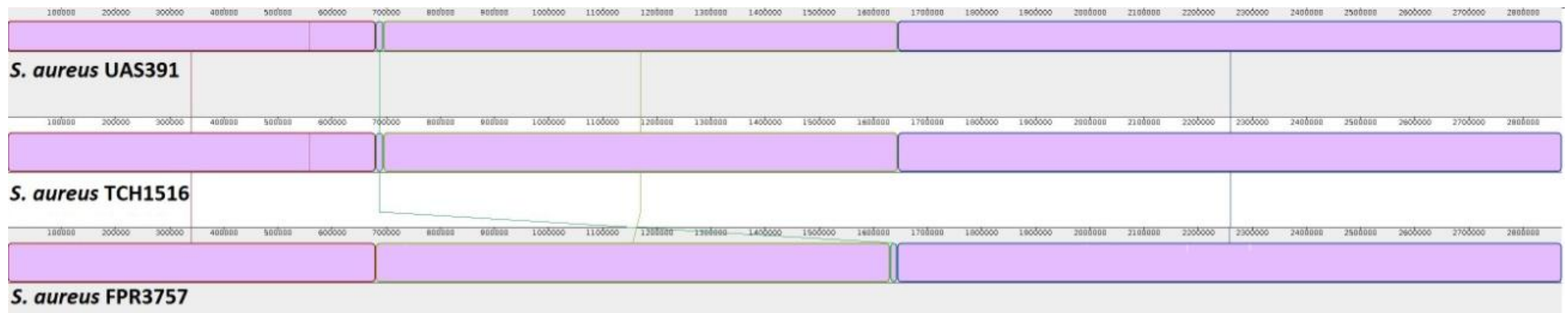

Supplement: Additional file 3: Figure S1. — Comparative genome alignment of USA300, TCH1516, FPR3757 and UAS391 shows high conservation between these genomes. Pink regions show homology and the vertical lines shows conserved blocks. The UAS391 ~13 kb genomic region (from 680369 bp to 693620 bp) is translocated to (1630711 to 1642611bp) in FPR3757. (PDF 116 kb) [file 12864_2015_1956_MOESM3_ESM.pdf]
