# Supplementary material for: Fatty acid kinase A is an important determinant of biofilm formation in Staphylococcus aureus USA300
Source: BMC Genomics. 2015 Oct 26;16:861. doi: 10.1186/s12864-015-1956-8 (PMC4623894; doi:10.1186/s12864-015-1956-8)
Supplement: Additional file 4: Figure S2. — Multiple alignment of fakA gene sequences in Staphylococcus aureus and Staphylococcus epidermidis. Unique nucleotide variation in SAUSA300_1119 of UAS391 encoding a fatty acid kinase fakA at position 1223940 nt, corresponding to nt 393 of the gene. The respective SAUSA300_1119 allele in UAS391 contains a non-synonymous SNP (Glu to Gly) as compared to strains TCH1516 and FPR3757. The Genbank accession numbers were indicated among these 50 strains. The first 45 strains represent S. aureus, the last 5 strains represent S. epidermidis. (PDF 897 kb) [file 12864_2015_1956_MOESM4_ESM.pdf]

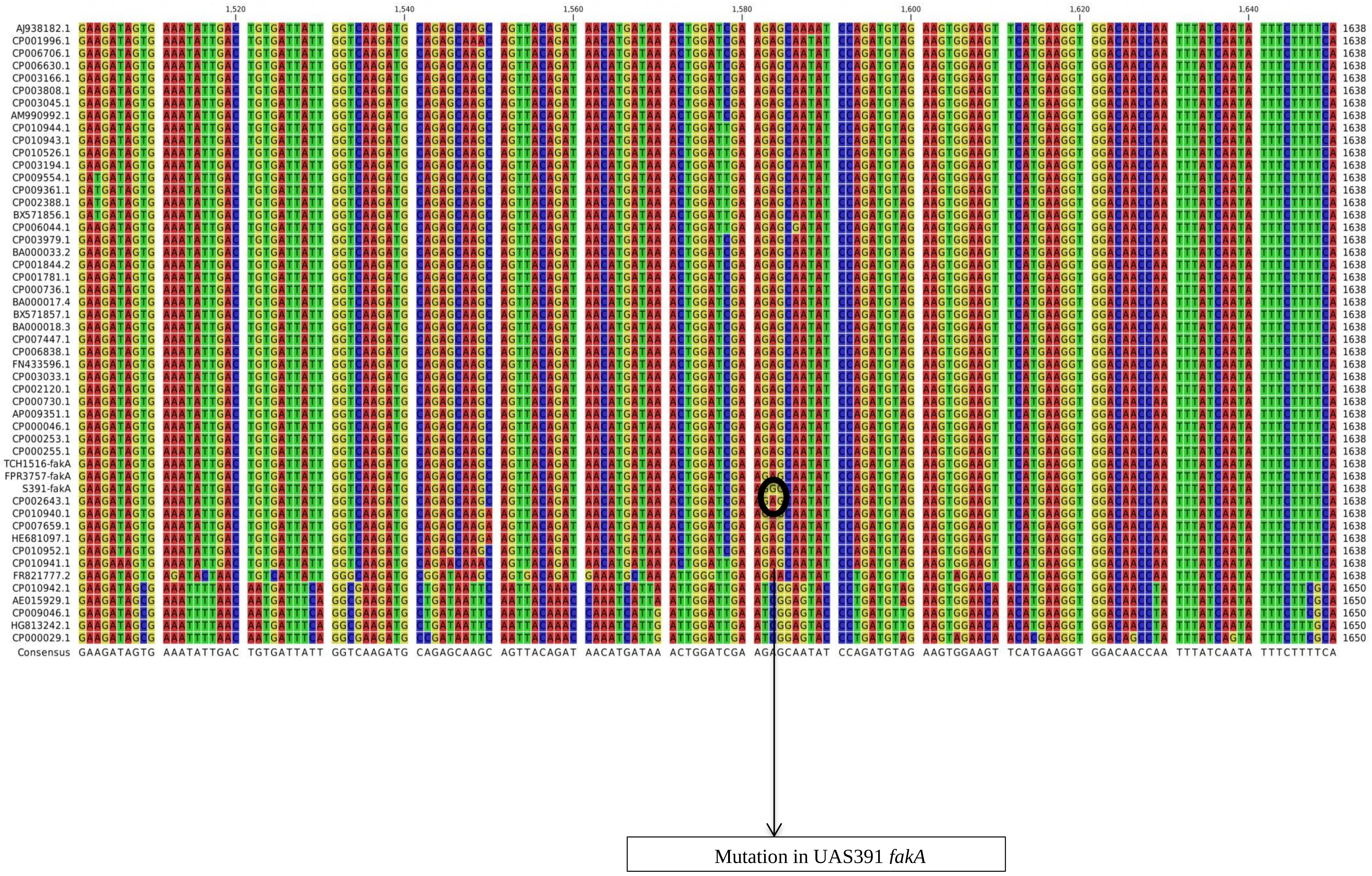
Mutation in UAS391 *fakA*
